# Antibacterial Designs for Implantable Medical Devices: Evolutions and Challenges

**DOI:** 10.3390/jfb13030086

**Published:** 2022-06-21

**Authors:** Huiliang Cao, Shichong Qiao, Hui Qin, Klaus D. Jandt

**Affiliations:** 1 Interfacial Electrochemistry and Biomaterials, School of Materials Science and Engineering, East China University of Science and Technology, Shanghai 200237, China; 2 Lab of Low-Dimensional Materials Chemistry, Key Laboratory for Ultrafine Materials of Ministry of Education, East China University of Science & Technology, Shanghai 200237, China; 3 Chair of Materials Science, Otto Schott Institute of Materials Research (OSIM), Friedrich Schiller University Jena, 07743 Jena, Germany; 4 Department of Implant Dentistry, Shanghai Ninth People’s Hospital, College of Stomatology, Shanghai Jiao Tong University School of Medicine, Shanghai 200011, China; 5 National Clinical Research Center for Oral Diseases, Shanghai 200011, China; 6 Shanghai Key Laboratory of Stomatology & Shanghai Research Institute of Stomatology, Shanghai 200011, China; 7 Department of Orthopaedics, Shanghai Jiaotong University Affiliated Sixth People’s Hospital, Shanghai 200233, China; 8 Jena Center for Soft Matter (JCSM), Friedrich Schiller University Jena, 07743 Jena, Germany; 9 Jena School for Microbial Communication (JSMC), Neugasse 23, 07743 Jena, Germany

**Keywords:** implantable antibacterial surfaces, polymicrobial infections, surface modification, biocompatibility, tissue integration, bacterial charging, cell-selective surfaces, antibiotic resistance, antimicrobials, protein adsorption

## Abstract

The uses of implantable medical devices are safer and more common since sterilization methods and techniques were established a century ago; however, device-associated infections (DAIs) are still frequent and becoming a leading complication as the number of medical device implantations keeps increasing. This urges the world to develop instructive prevention and treatment strategies for DAIs, boosting the studies on the design of antibacterial surfaces. Every year, studies associated with DAIs yield thousands of publications, which here are categorized into four groups, i.e., antibacterial surfaces with long-term efficacy, cell-selective capability, tailored responsiveness, and immune-instructive actions. These innovations are promising in advancing the solution to DAIs; whereas most of these are normally quite preliminary “*proof of concept*” studies lacking exact clinical scopes. To help identify the flaws of our current antibacterial designs, clinical features of DAIs are highlighted. These include unpredictable onset, site-specific incidence, and possibly involving multiple and resistant pathogenic strains. The key point we delivered is antibacterial designs should meet the specific requirements of the primary functions defined by the “*intended use*” of an implantable medical device. This review intends to help comprehend the complex relationship between the device, pathogens, and the host, and figure out future directions for improving the quality of antibacterial designs and promoting clinical translations.

## 1. Introduction

It was estimated that over 500,000 types of medical devices, such as dental implants, vascular graft/endograft, orthopedic prosthetics, catheters, etc., are currently marketing globally for medical applications [1]. Every year, there are about 10,000,000 dental implants and more than 1,000,000 cardiovascular electronic devices inserted around the world [2,3]. It has been estimated that 100 million urinary catheters are sold worldwide each year [4]. As the population of the aged increases, procedures for implantable medical devices are expected to increase rapidly in the coming years. In the United States of America (USA), the primary total knee arthroplasty (TKA) is going to grow by 85%, to 1.26 million procedures by 2030 [5]. In Germany, by 2040, the total number of TKA is expected to increase by 45% to over 244,000 procedures; and the incidence rate of total hip arthroplasty (THA) is projected to increase to 437 per 100,000 inhabitants [6]. In the United Kingdom, the volume of hip and knee joint replacement is expected to increase by almost 40% by 2060 [7]. Bacterial infections are one of the most frequent and severe complications associated with the clinical application of implantable medical devices [1]. It was reported that device-associated infections (DAIs), including ventilator-associated pneumonia, catheter-associated urinary tract infection, and central-catheter-associated bloodstream infection), accounted for approximately 26% of all healthcare-associated infections (HAIs) in the USA [8]. The annual number of HAIs in European Union countries is about 3.2 million, including 37,000 registered mortalities [9]. The financial burden for the treatment of a DAI is also extraordinarily high. For instance, the average revision costs in the USA for infected hip and knee arthroplasty were approximately USD 80 and 60 thousand, respectively [10]. Additionally, by 2030, the estimated combined annual hospital costs related to arthroplasty infection will rise to USD 1.85 billion in the USA alone [11]. This urges the world to develop instructive prevention and treatment strategies for DAIs.

Accordingly, fundamental research on the development of various antibacterial surfaces has dramatically increased in recent years. Screening for “*antibacterial surface*” or “*antibacterial coating*” in the topic of the articles included in the Web of Science (*www.webofscience.com*; accessed on 14 February 2022) can hit more than 50,000 records between the years 1996 and 2021. Around 80% of these records were published during the last decade (between 2012 and 2021), and over 67% of them were published during the last five years (between 2017 and 2021), identifying a boom in developing antibacterial surfaces or coatings. Developing antibacterial surfaces for implantable medical devices also is currently a hot direction among the Chinese communities focusing on biomaterials science and engineering. Typical designs published in the first half of 2022 include copper-bearing titanium [12], surface charge and wettability control in lysozyme [13], light-activatable carbon monoxide gas generation by triiron dodecacarbonyl loaded polydopamine [14], clickable peptide engineered surface [15], calcium-doped titanium targeting blood protein adsorption [16], puncture and ROS (reactive oxygen species) release by nanorod zinc oxide patterns [17], light-stimulated ROS generation by rare-earth elements-doped titanium dioxide coating [18], on-demand antibiotics release by responsive polymers [19,20], and bacteriophage-modified alginate hydrogels [21]. This trend demonstrates that the academic community has already realized the urgency of solving the DAI problem, whereas only a limited number of these innovations have entered clinical applications or clinical studies around the world. A very small number of registered records (we found merely eight) concerning antibacterial surfaces were found in *ClinicalTrials.gov* (accessed on 22 May 2022) by searching for “*device infection*” in the “*Condition or disease*” field. As shown in Table 1, silver in metallic or ionic forms is the most popular active ingredient in developing antibacterial medical devices. Currently, a handful of antibacterial surfaces have been branded for clinical uses, which are commonly silver-based and normally custom-made (available on request). These include *Acticoat* using magnetron sputtering synthesized nanosilver coatings for wound care [22], *MUTARS* prosthesis reducing infections by electroplating a metallic-silver surface, METS prosthesis acting against pathogenic bacteria by absorption of ionic silver to anodized titanium implants [23], *PorAg* prosthesis taking advantage of a controlled electrochemical reaction (do not directly release silver ions) in a titanium-silver alloy for disinfection [23], and *PROtect* nails administrating gentamicin for prevention of infections in complex open fractures [24]. These commercial promotions have set examples for the development of antibacterial surfaces for implantable medical devices (here we coin them “*implantable antibacterial surfaces*”); however, it is still a challenge to improve the quality and efficiency of translational research over those “*antibacterial surface*” or “*antibacterial coating*” reports.

Herein we firstly analyze the cases associated with device-associated infections (DAIs) by highlighting the clinical features and challenges in DAIs prevention and treatment, then present the state-of-art research by identifying the evolutions in developing antibacterial surfaces for implantable medical devices, i.e., implantable antibacterial surfaces and, finally, illuminate the flaws in reporting of the findings in fundamental researchers to advance the development and translation of innovative designs against bacterial infections and promote the success of implantable medical devices.

## 2. Clinical Features of Device-Associated Infections

### 2.1. Site-Specific Incidence

Infection is a common and frequent complication associated with all types of biomedical materials, despite the infection rate varying greatly among different intended uses of various implantable devices (Table 2) [25,26,27,28,29,30,31,32,33,34,35,36,37,38,39,40,41,42,43,44,45,46,47,48,49,50,51,52,53,54,55,56,57,58,59,60,61]. Orthopedic implants, such as the ankle, hip, knee, elbow, shoulder, and finger joint prosthetics, are made of metals (titanium alloys, stainless steel, cobalt-chromium alloy, etc.) and are expected to serve long periods (>10 years) in patients’ bodies. Infections of these devices are extremely troublesome [1]. Ankle arthroplasty has higher infection rates (2.4–8.9%) than hip (0.4–2.4%) and knee (1–2%) arthroplasty, although they are normally made of the same materials (Table 2). This is remarkably related to wound dehiscence (or prolonged drainage) developed due to the frail soft tissue surrounding ankles and increased chance of delayed wound healing following ankle arthroplasty [26,62]. The infection situation will be even more serious in revision cases. For example, the incidence of infection for primary hip and knee arthroplasty is around 2% (Table 2), yet this will be possibly as high as 12% and 22% for revision hip and knee arthroplasty, respectively [63]. Moreover, the number of infection cases is expected to increase progressively because the number of arthroplasty surgeries is going to grow in the coming years. In Taiwan, China, for instance, a total of 728 hip and knee infection cases were recorded in 2013 and this number was expected to increase markedly to over 3500 by 2035 [10]. Not only these metallic implants are connected to bacterial infection, but also polymer devices are susceptible to this complication (Table 2). Examples include breast implants, vascular graft/endograft, cardiovascular electronic devices, and cochlear implants, which are made of silicone, polytetrafluoroethylene, plastics, or Teflon, and have infection incidence high up to 10.2% [32], 6% [33], 7% [37], and 8% [40], respectively. Additionally, the DAIs may occur due to the device design. As in brain stimulation implants, the battery of the pulse generator should be replaced typically every 2 years, and such multiple replacements increase the risk of DAIs [46]. Furthermore, the incidence of infection is highly determined by the site a device is placed in. As shown in Table 2, the infection rates in urinary catheters (up to 13.7 cases per 1000 catheter-days), cerebrospinal fluid shunts (27%), internal fixation devices (32%), and dental implants (47%) are high. This is because these devices are highly challenged by bacterial adhesion and biofilm formation during their insertion and the subsequent service period. For example, urinary catheters provide routes for the entry of pathogenic bacteria, increasing the risk of acquiring infections [51]. Investigations of the bacterial sources in infected shunts also demonstrate that a majority of harmful microbes gained entry from the skin of the patients themselves [64]. The risk of complications in fixation of fractures is highly in connection to the low blood supply and elder people are susceptible to infection [59]. Additionally, there are more than 500 bacterial species associated with commensals or pathogens within the oral cavity [65]. This situation makes the prevention of infections in dental implants extremely complicated. The reported incidence rates for dental implants serving of over 3 and 5 years are 9.25% and 9.6%, respectively, and this rate for implants with service periods of over 10 years is up to 26% [61]. More importantly, the prevalence of the pathogenic strains is also associated with specific anatomical locations. Although *Staphylococcus* spp. is the most prevalent microbe associated with all types of bacterial infections, other pathogens can be involved in specific sites. Gram-negative microbes are involved in 10–40%, 20%, and 35–55% of vertebral, trauma/fracture, and foot/ankle-related infections [66]. Additionally, 15–30%, 20–30%, and 30–80% of polymicrobial infections occur in vertebral, trauma/fracture, and foot/ankle, respectively [66]. Different bacterial strains may have different metabolisms and pathogenic mechanisms that require specifically tailored treatments. This is especially critical to cure infections involving multiple pathogenic strains; as a result, developing an all-around antibacterial solution for all medical devices is hardly possible.

### 2.2. The Unpredictable Onset

Device-associated infections become even stickier because of those host-specific, transient, or resident factors (Table 3) [67,68,69,70,71,72,73,74,75,76,77,78,79]. The onset of DAI is not predictable, it can onset years after implantation (Cases 1 through 6 in Table 3). The soft tissue envelope around an implant likely degenerates with aging and can be disrupted by an occasional scratch, which may have promoted the infection of an alloplastic chin implant 45 years after placement [67]. Breast implants significantly risk bacterial contamination from hematogenous spread of distant antecedent infections. It was reported that the *Achromobacter xylosoxidans* (lives in wet soil) from a chronic footsore and *streptococcus viridans* (lives in the oral cavity) from recurrent periodontitis can cause infection of breast implants even 7 and 25 years after the implantation [68]. *Staphylococcus epidermidis* (*S. epidermidis*) can colonize various biomedical implants and escape from the immune clearance and antibiotic treatments, hence possibly causing symptom-free (such as pain, redness, or fever) chronic infection lasting even for 30 years before being identified by clinical approaches [69]. *Cutibacterium acnes* (previously known as *propionibacterium acnes*), a common conjunctival inhabitant, are slow-growing, anaerobic Gram-positive rods, and can manifest several years or even decades before leading to late infections in orbital implants made of silicone or tantalum [70,71]. The sources of the pathogens of the DAIs can be host-specific (Cases 7 through 9 in Table 3). DAIs can be initiated by acute illness (e.g., diarrhea developed during a holiday journey [31]), penetration of contaminated water during participating in outdoor activities [45], or even when the patients play with their pets (bacterial contamination from zoonotic sources) [72]. Moreover, the occurrence of DAIs is commonly associated with a compromised immune system in the hosts (Cases 10 and 11 in Table 3). Methotrexate, a folate antagonist, can affect neutrophil chemotaxis and induce apoptosis of T cells and reactivation of opportunistic pathogens; hence chronic treatment of rheumatoid arthritis with this kind of drug significantly increases the risk of infections around the battery for brain stimulation [73]. *Nocardia nova* is a common environmental pathogen and rarely affects immunocompetent hosts; however, this species successfully colonized a tibia implant placed in an immunocompetent patient [74]. *Listeria monocytogenes*, a common organism associated with unpasteurized dairy products (e.g., deli meats and unpasteurized cheeses), can induce a periprosthetic joint infection in a patient with a history of diabetes mellitus, asthma, and psoriatic arthritis [75]. *Anaerobiospirillum succiniciproducens*, a common settler in the gastrointestinal tract of cats and dogs, can induce a prosthetic hip joint infection in an immunocompromised patient [76]. DAIs are normally initiated by bacterial seeding and as a result tissue integration will be impaired quickly; however, some cases failed to identify any organism by cultures [77,78] and tissue integration was intact after being infected [79]. These situations add difficulties to the prevention, diagnosis, and treatment of DAIs.

### 2.3. Diversity of Relevant Pathogens

Infections associated with medical devices with the same intended use (the same device category) but placed in different individuals are possibly connected with different bacterial strains. As shown in Table 3, the infection of breast implants can result from *achromobacter xylosoxidans* (Gram-negative rod) [68], *streptococcus viridans* [68], and *salmonella serogroup C* [31], or *Pasteurella canis* [72]. Polymicrobial infections become more prevalent in DAIs [66,80]. Even a single infection in a specific individual often has a polymicrobial composition [81]. Multispecies including the rare *Cupriavidus pauculus* species were isolated in an infection associated with the generator for brain stimulation [45]. Since the bacteria associated with an infection of a medical device may have diverse morphologies and arrangements, an effective antibacterial strategy must be capable of eliminating multiple pathogenic species. *Cocci* cells (spherical bacteria) range from 0.5 to 2.0 µm in diameter, rods are approximate 0.5–1.0 µm in width and 1–10 µm in length, and spiral bacteria are up to 20 µm in length and 0.1–1 µm in diameter [82]. Moreover, bacterial morphology varies with the growth environments (medium, surfaces, etc.) and growth phase (normally smallest in the logarithmic phase) [83,84]. These facts add additional difficulties to developing a competent antibacterial surface for implantable devices. On account of these features of DAIs, antibacterial surfaces only have a pore-size-based cell selectivity [85], or those peptide-loaded surfaces merely have specific actions to Gram-positive or Gram-negative strains [86] and are not likely adequate to prevent infection of implantable medical devices.

### 2.4. Prevalence of Antibiotic Resistance

The uses of internal implants in humans are safer and more common since sterilization methods and techniques were established at the end of the 19th century [87], and the commercialization of antibiotics especially penicillin in the first half of the 20th century [88]. Antibiotics have become an integral component of contemporary biomedical practice, producing a serious follow-up threat: antibiotic resistance in bacteria [89,90]. Clinical cases in orthopedic practice have shown that infections of antibiotic-resistant bacteria, such as methicillin-resistant *Staphylococcus aureus* (MRSA), are closely related to high morbidity and mortality [91]. Antibiotic resistance in bacteria even multidrug-resistant (MDR) bacteria is now a worldwide challenge [91]. Antibiotic-resistant infections were frequently reported all over the world, including in both developing and developed countries (Table 4) [92,93,94,95,96,97,98,99,100,101,102,103,104,105,106,107,108,109,110]. During an infection, *Staphylococcus aureus* (*S. aureus*) often forms biofilms on implantable devices, which dramatically increases the ability of the species to acquire resistance via horizontal plasmid transfer [111]. This is why *S. aureus* has high rates of resistance. As shown by the typical cases reported in recent years (Table 4), MRSA has become the most common strain causing infections of various implantable medical devices, including cardiac devices [93,95,99,103,106], orthopedic prosthetics [96,97], cochlear implants [98], breast implants [100], laryngeal implants [101], and stent grafts [109]. In addition, there is an alarming increase in antibiotic resistance in other strains, such as *Acinetobacter baumannii* [92], *Mycobacterium chelonae* [94], *Enterobacter cloacae complex* [102], *S. epidermidis* [104,110], *Klebsiella pneumoniae* [105], *Staphylococcus haemolyticus* [107], and *Staphylococcal endophthalmitis* [108], are also involved in various resistant DAIs. Those resistant DAIs impacted patients have to experience prolonged hospital stays, bear high medical costs, and risk increased mortality (references in Table 4). Antibiotic recalcitrance is a worldwide threat that likely causes substantial global economic costs ranging from USD 21,832 per individual case to over USD 3 trillion in gross domestic product (GDP) loss by 2050 [112]. In the USA alone, at least 2 million infections and 23,000 deaths per year were caused by antibiotic-resistant bacteria, costing USD 55–70 billion [90]. Currently, antibiotic-loaded materials are important complements to modular medical practices for the prevention of recurrent infections in various medical devices, such as wound dressings, bone cement, bone plates, nails, or prostheses [24,113,114]. However, applications of these surfaces in “*uninfected tissues*” to prevent DAIs should be careful and in strict guidance, because the prolonged release of prophylactic antibiotics possibly contributes to arising resistant mutants [115]. Silver-based surfaces also have attractive efficacy in the prevention of DAIs [116], improper use of this material may also pose bacterial-resistant problems [117,118]. In addition, pathogenic bacteria have many defensive actions resistant to antimicrobial challenges [91,119,120]: (a) express polymer biofilms to protect themselves from antibiotic attacks; (b) remodel their outer surface to reduce antibiotic uptake; (c) synthesize precursors to modify the target of antimicrobials; (d) produce enzymes to detoxify dangerous drugs. Therefore, antibacterial surfaces, especially those release-killing ones, should be designed to bypass these actions of bacterial cells.

## 3. Innovative Designs to Mitigate Device-Associated Infections

**Figure 1:**
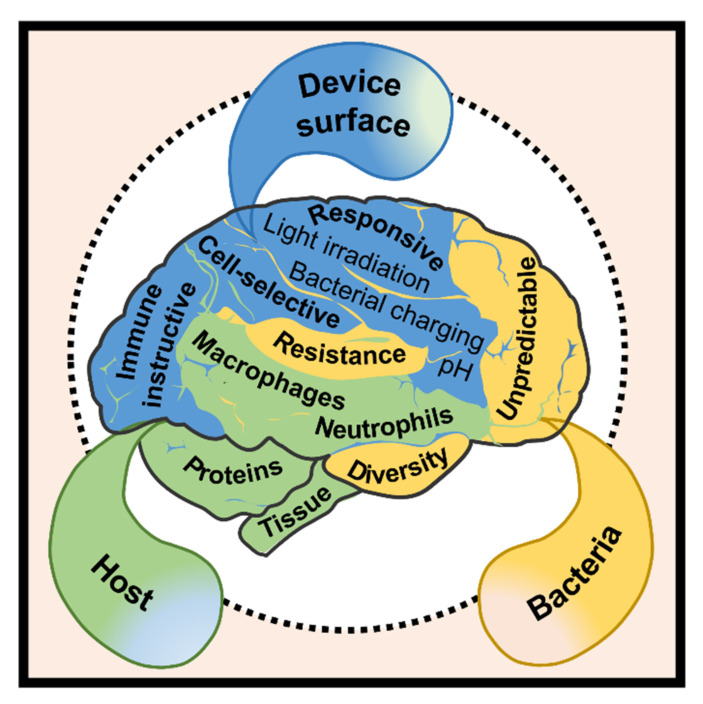
Rethinking the interplay among device surface, host, and pathogen.

Based on the reports we screened, the innovations of implantable antibacterial surfaces can be categorized into four groups, namely surfaces with prolonged or cell-selective bactericidal efficacy and responsive or immune-instructive surfaces. Prolonged antibacterial efficacy (or long-term antibacterial activity) can be realized by taking advantage of the degradation or surface structures of the supporting materials for orchestrated release or immobilizing the antimicrobials yielding contact-killing surfaces. Cell selectivity of antibacterial surfaces can be obtained by doping of multifunctional metals, the combinational release of ingredients that are respectively good for antibacterial and tissue integration promotion or applying the electrochemical reactions evoked by the host’s physiological fluids to recognize bacteria from mammalian cells. Responsive antibacterial surfaces can deliver services over the stimulation of external light irradiation, or internally by the bacterial charge or bacterial infection-associated pH shifts. Since the key players (neutrophils and macrophages) in the immune system can be regulated by proper surface chemistry, topography, wettability, or stiffness, immune-instructive antibacterial surfaces are expected to be produced by control of these parameters. These evolutions in the development of implantable antibacterial surfaces help us rethink those complex interactions among device surface, host, and the pathogen (Figure 1), advancing the solution of DAIs.

### 3.1. Prolonged Antibacterial Efficacy

**Figure 2:**
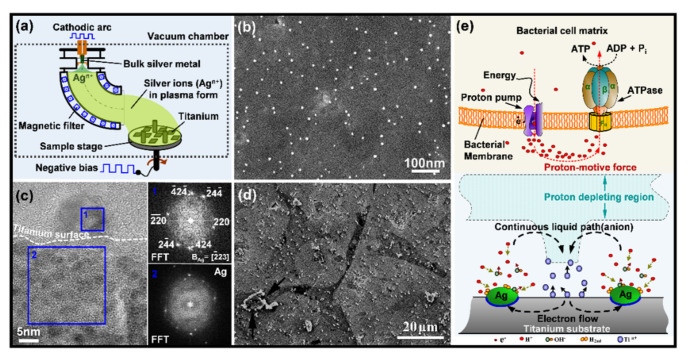
Contact killing of silver nanoparticles synthesized and immobilized on titanium by ion implantation: (**a**) schematic representation of the silver plasma immersion ion implantation and deposition (Ag PIII&D) process; (**b**) SEM image of the silver nanoparticles synthesized and immobilized on titanium by Ag PIII&D under a 30 kV bias for 30 min; (**c**) cross-sectional TEM of the silver nanoparticles synthesized and immobilized on titanium by Ag PIII&D, with corresponding fast Fourier transform patterns (FFT, 1 and 2) inserted; (**d**) SEM image of the Staphylococcus aureus cells cultured on an Ag PIII&D treated (treated for 30 min under a 30 kV bias) titanium for 24 h at 37 °C with a bacteria concentration of 10^8^ CFU/mL; (e) possible antibacterial mechanism of the Ag PIII&D treated titanium; (**b**, **d**, and **e**) reused with permission from Elsevier [143]; (**c**) reused with permission from American Chemical Society [144].

As shown by Table 3 and Table 4, the latent period of a DAI can be days after implant placement [92,94,105,108,109,110], years after the surgery [78,79,93,95], or even decades later [67,68]. This feature of DAIs lays the basis for the development of antibacterial surfaces with long active durations. As shown by the representative reports on the development of “*long-term*” antibacterial surfaces (Table 5) [121,122,123,124,125,126,127,128,129,130,131,132,133,134,135,136,137,138,139], various ingredients such as commercial antibiotics (tigecycline, vancomycin, amoxicillin, etc.) [121,132], metals or metal ions (silver, copper, or zinc) [124,125,129], and other drugs [127,128] were taken to equip implantable biomaterials (titanium, silicone, ceramics, etc.) with prolonged antibacterial efficacy, ranging from days [127,131,133] to months [122,134]. Extending the release period of the antimicrobials is currently a major pathway leading to “*long-term*” antibacterial surfaces. Calcium phosphate cement (CPC) has proved an effective carrier to retain vancomycin (effective for the treatment of MRSA) to local sites [140,141], ensuring the antibiotic has a 24-week release profile in vivo [122]. Proper antibiotic concentration is a key factor that determines the mechanical strength of vancomycin-impregnated CPC and influences the effective antibacterial period of the composite [140]. Electrochemical oxidation, namely micro-arc oxidation (also known as plasma electrolytic oxidation) and anodic oxidation, is a well-known class of approaches that can produce porous surface layers on implant materials and, in the meantime, load antibacterial agents on the material’s surface. Shivaram et al. demonstrated that the silver loaded in an anodized titanium substrate had a release period of a minimum of 6 months [134]. The titanium substrates were fabricated with 25 vol% porosity by using a powder-based additive manufacturing technique [142]. Then electrochemical anodization was applied to the porous titanium in a hydrofluoric acid electrolyte to produce a surface layer of nanotube arrays with a thickness of 375 ± 35 nm and diameter of 105 nm ± 30 nm, which facilitated the loading of silver from a 0.1 M silver nitrate (AgNO_3_) solution via direct current deposition [134]. After heating at 500 °C, tightly adhered silver particles with a coverage of 13.5% were detected on the nanotube-structured surface. The 27-week cumulative release profiles demonstrated that silver release was within 10 ppm (mg/mL), which ensured good early-stage osseointegration of the porous titanium implants, along with good antibacterial activities [134]. Micro-arc oxidation is another technique that can produce a porous titanium surface which may facilitate the control of antimicrobial release. Very recently, Tsutsumi et al. reported that silver and zinc load micro-arc oxidation layer on titanium exhibited excellent activity against *Escherichia coli* (*E. coli*) after a six-month immersion in physiological saline [124]. Another way to prolong the effective period of antimicrobials is to immobilize (or embed) them in the non-degradable implant surfaces and prevent release. Cao et al. developed a silver plasma immersion ion implantation and deposition (Ag PIII&D) procedure to in situ synthesis and immobilize silver nanoparticles (Ag NPs) on titanium surface [143]. The process is generally carried out in a vacuum chamber of about 2.5 × 10^−3^ Pa and takes a pure silver rod (10 mm in diameter) as a cathode to produce cathodic arcs, which serve as sources of positively charged silver ions (Ag^n+^) (Figure 2a). The silver arcs are filtered by a curved magnetic duct to remove the macro-particles produced. The energetic silver ions in a plasma form are accelerated and injected in a non-line-of-sight manner onto the titanium surfaces, which are negatively biased by a pulsed high voltage synchronizing with the cathodic arc current under a certain frequency. The positively charged silver ions become neutral atoms when they reach the titanium surfaces. As the process continues, the neutral atoms are further condensed and nanoparticles precipitate. By using this process, well-distributed Ag NPs were synthesized and immobilized on titanium. Figure 2b shows a group of Ag NPs average of 5 nm in diameter was produced by Ag PIII&D under a 30 kV bias for 30 min. Cross-sectional TEM images also confirmed that those Ag PIII&D produced nanoparticles were metallic silver (Figure 2c) [144]. The antibacterial efficacy of these nanoparticles was found independent of silver release [143]. As shown in Figure 2d, the grain boundaries of the titanium substrate were exposed after the material (Ag PIII&D treated for 30 min under a 30 kV bias) have defeated the colonization of *S. aureus* (cultured for 24 h at 37 °C with a bacteria concentration of 10^8^ CFU/mL), indicating the antibacterial efficacy of those immobilized Ag NPs is associated with the corrosion of the titanium substrate. Since the standard electrode potential of titanium, −1.63 V, is more negative than that of silver at 0.80 V, the Ag PIII&D treated titanium surface embedded with a proper number of well-distributed Ag NPs likely evoked micro-galvanic corrosion, in which the cathodic reactions on Ag NPs may establish proton depleted regions on the titanium surface that possibly disrupt the proton electrochemical gradient (i.e., proton-motive force) in the intermembrane space of the microbes and get them killed (Figure 2e) [143]. Followed-up studies demonstrated that Ag PIII&D treated titanium implants have a long activity duration (60 days, the longest time point studied) against bacterial colonization [138]. The effectiveness of this micro-galvanic-associated antibacterial mechanism in copper-bearing stainless steels was also evidenced recently [145]. It was found that the contact killing of copper-bearing stainless steel was manipulated by the potential difference of the microdomains (the copper-rich phase and the matrix) in the material, which also produced proton depletion in bacteria and as a result cell death [145].

These studies provide insights into solving the problem of DAIs; however, they are still far from meeting all the clinical requirements. This applies, especially for those late hematogenous cases, which may suddenly come years or decades after surgeries [68]. At present, there is no clear definition of the time of effectiveness of “*long-term*” antibacterial surfaces. Our opinion on determining “*long-term*” is to clarify the “*intended use*” of the surface first and take into account the time for a specific tissue repair process. The skin healing process consists of ordered stages, which are inflammation (15 min to 6 days), proliferation (2–3 days to 2–3 weeks), and maturation (3 weeks to 2 years) [132]. In this respect, we believe that the effective period for a “*long-term*” antibacterial wound dressing should exceed 3 weeks. Generally, the healing of a bone fracture involves three distinct but overlapping stages, i.e., the early inflammatory period (a few hours to days), the repairing period (weeks), and the late remodeling period (months to years) [146,147]. As a result, the typical time for a new bone to achieve adequate strength is 3 to 6 months [146]. Therefore, we believe the effective period for “*long-term*” antibacterial bone implants should exceed 3 months, and this time required for elder patients should be longer because their bone healing process is relatively slow. Although studies on “*long-term*” antibacterial surfaces are increasing these years, most of the reports did not consider the “*matching*” problem between antibacterial duration and tissue integration, yielding barriers to translational research. Since the incidence of DAI is site-specific, this aspect should be considered in future studies.

### 3.2. Response to pH Shifts

**Figure 3:**
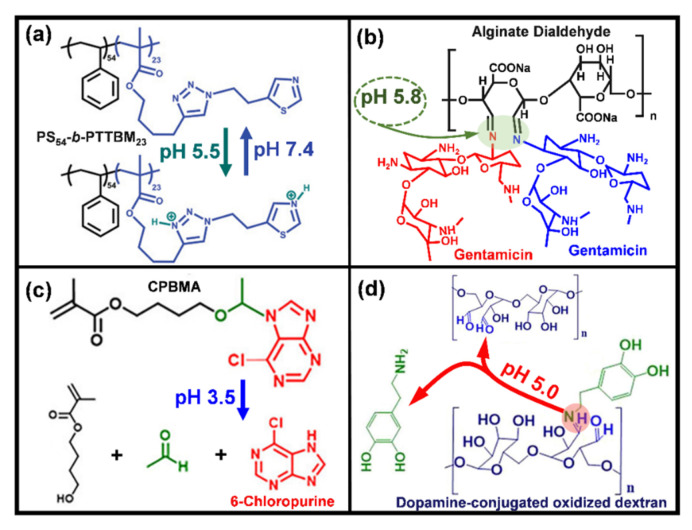
Typical methods toward pH-responsive surfaces: (**a**) protonation of polystyrene-*b*-poly(4-(1-(2-(4-methylthiazol-5-yl)ethyl)-1*H*-1,2,3-triazol-4-yl)butyl methacrylate) (PS_54_-*b*-PTTBM_23_) at acidic pH levels and increase of the positive charge density on the surfaces [151]; (**b**) breaking the Schiff base bonds between antibacterial gentamicin and alginate dialdehyde by acidic environments [157]; (**c**) hydrolyzation of the hemiaminal ether linkage of antimicrobial 6-Chloropurine in 4-(1-(6-chloro-7H-purin-7-yl) ethoxy) butyl methacrylate (CPBMA) by mild acidic conditions [158]; (**d**) destruction of dopamine-conjugated oxidized dextran polymer to release the contained silver nanoparticles by disintegration the Schiff base structures in the polymer [160]. (**a**,**c**) reused with permission from John Wiley and Sons and American Chemical Society, respectively; (**b**,**d**) reused with permission from Elsevier.

It is known that the pH shift is a common phenomenon of bacterial infections [148,149,150], laying the basis for the development of pH-responsive antibacterial surfaces. The antibacterial activity of pH-responsive films or coatings can be triggered by the protonation or deprotonation of their ionic groups. The thiazole and triazole groups, for example, in polymer PS54-b-PTTBM23 (on porous polystyrene surfaces) can be protonated under acidic pH levels, which increased the positive charge density on the materials surface to act against bacterial adhesion (Figure 3a) [151]. In addition, the killed bacteria can be further removed by increasing the pH levels (pH 7.4, for instance), which induced deprotonation of the thiazole and triazole groups in the materials [151]. Normally, pH-responsive surfaces are designed for the controllable release of antibacterial agents by manipulating the materials’ pH-associated swelling or shrinking processes. By shifting the environmental pH, the protons of the carboxyl repeat units in poly(methacrylic acid) can be removed to make the material swell, which can control the release of antibacterial drugs [152]. In this manner, Wei et al. developed a pH-responsive surface capable of loading bacteriolytic lysozyme at acidic pH levels and releasing it under neutral or basic pH [152]. A pH-sensitive fibrous membrane was also developed to control the release of antibacterial gatifloxacin hydrochloride and silver nanoparticles [153]. The backbone (hydrophobic)-attached amino groups (weak basic moieties) of chitosan adapt to a deprotonated state above pH 6 while becoming protonated and positively charged at low pH, demonstrating a pH-dependent extension of the colloid chains and consequently swelling of the material [154]. Accordingly, chitosan was crosslinked with hydroxypropyl methylcellulose and 2-hydroxypropyl-β-cyclodextrin to produce a superabsorbent hydrogel for controllable delivery of antibacterial 3,4-dihydroxy cinnamic acid in response to pH changes [154]. Similarly, the structure of keratin hydrogel was reorganized by manipulating the protonation and deprotonation process of carboxyl groups in the material, yielding pH-dependent shrinking and swelling at low and high pH levels, respectively [155]. This behavior of the keratin hydrogel was taken to control the release of biocidal zinc oxide nanoplates in a pH-dependent manner, which can be a potential therapy response to a bacteria-contaminated media with biased pH and treatment of chronic wounds [156].

In addition, acid-labile bonds can also be used to program the release of antibacterial agents. Antibacterial gentamicin was conjugated with an alginate dialdehyde Schiff base reaction between the aldehyde groups (-CHO) and amino groups (-NH2) from the polymer, and was released due to the acidic environment triggered the disintegration of the Schiff base bonds (Figure 3b) [157]. Similarly, antimicrobial 6-Chloropurine was conjugated to 4-(vinyloxy) butyl methacrylate (VBMA) to produce 4-(1-(6-chloro-7H-purin-7-yl) ethoxy) butyl methacrylate (CPBMA), which contained a hemiaminal ether linkage can be hydrolyzed in mildly acidic conditions and allowed the controllable release of the antibacterial agent (Figure 3c) [158]. Moreover, pH-induced material structural evolutions, such as degradation, disintegration, and conformational changes, are also applied for the controllable delivery of biocides. Polyacetal-based polymers are degradable under acidic pH levels and possess a relatively low critical solution temperature (LCST) which allows wettability control by shifting the temperature (between LCST and room temperature) [159]. On account of this, a film-forming triple polymer-gel matrix containing polyacetal-based polymer was prepared by De Silva et al. to control the topical release of silver sulfadiazine, which was highly efficient against wound pathogens, such as *S. aureus*, *Pseudomonas aeruginosa* (*P. aeruginosa*), and *Candida albicans* (*C. Albicans*) [159]. The Schiff base structure between the amino groups (-NH_2_) in dopamine and the aldehyde groups (-CHO) in oxidized dextran can be formed at pH 7.0 under the protection of nitrogen (N_2_) [160]. The Schiff base bonds were disintegrated due to exposure to acidic bacteria-infected diabetic wounds, which was the mechanism used by Hu et al. to control the release of antibacterial silver nanoparticles by dopamine-conjugated oxidized dextran polymers (Figure 3d) [160]. The pH-induced conformational changes in silk fibroin (in a nanocapsule structure) were also applied to control the delivery of eugenol, which exhibited strong efficacy against both Gram-positive *S. aureus* and Gram-negative *E. coli* [161].

### 3.3. Response to Bacterial Charging

Membrane-bound respiratory electron transfer in bacteria plays a critical role in the synthesis of adenosine triphosphate and bacterial maintenance [162]; therefore, it can be a potential target for antibacterial surfaces. Extracellular electron transfer is a general mechanism required for bacterial growth [163,164,165,166]. The microbial cell envelope is not electrically conductive; hence bacteria have evolved strategies to exchange electrons with extracellular substances [167], including direct electron transfer via physical contacts (through the bacterial envelope or pili) between a microbe and a material surface, and redox-active compounds mediating electron shuttle between bacteria and the material’s surface serve as electron acceptors [168].

**Figure 4:**
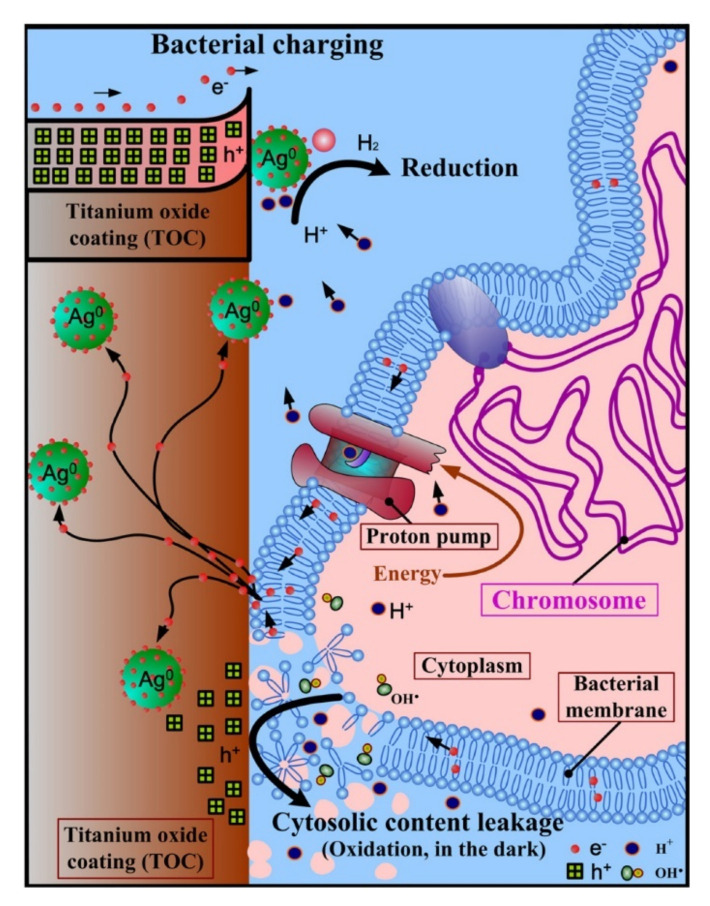
Silver nanoparticle decorated titanium oxide coating acting against bacterial colonization by taking advantage of extracellular electron transfer in bacteria: collection and storage of bacteria-extruded electrons on the immobilized silver nanoparticles (“bacterial charging”), accumulation of valence-band hole (h^+^) at the titanium oxide side of the silver–titanium oxide boundaries, and disruption of bacterial cell walls (cytosolic content leakage) by those accumulated valence-band holes (oxidation) [169]. Reused with permission from Elsevier.

Accordingly, Cao et al. proposed to construct antibacterial coatings targeting the extracellular electron transfer process in pathogenic bacteria (Figure 4) [169,170]. Ag NPs in various sizes (4–25 nm) were in situ synthesized and immobilized onto plasma-spraying-prepared titanium oxide coatings by manipulating the atomic-scale heating effect in silver plasma immersion ion implantation. The antibacterial efficacy of the resulting composite coatings was dependent on the particle size and interparticle space of the immobilized silver, i.e., large particles (5–25 nm) induced fatal cytosolic content leakage and lysis of both Gram-negative and Gram-positive bacteria while small ones (~4 nm) did not [169]; and a relatively large interparticle space was superior to a small interparticle space is disrupting the integrity of the adherent bacterial cells [170]. Similar results were also reported in follow-up studies by using silver nanoparticles decorated with tantalum oxide coatings [171,172]. By using plasma spraying, graphene nanomaterials decorated with titania coatings were prepared for antibacterial applications [173]. The coatings can collect the electrons extruded by adherent bacterial cells due to the rectification of the Schottky-like graphene-titania boundaries [173]. In vitro evidence showed that cobalt-titanium dioxide and cobalt oxide (CoO or Co_3_O_4_)-titanium dioxide nanoscale heterojunctions can downregulate the expression of respiratory gene levels in bacteria and cause oxidative damage to bacterial surfaces [174]. In another study, Wang et al. also found that the antibacterial efficacy of tungsten-incorporated titanium dioxide coatings (prepared by micro-arc oxidization) was related to their strong capability in the storage of bacteria-extruded electrons and accumulation of sufficient valence-band holes inducing oxidative damages to the microbes [175]. These findings have opened up new avenues for taking advantage of the intrinsic feature of biological systems to design and control the antibacterial actions of biomaterials.

### 3.4. Response to Light Irradiation

**Figure 5:**
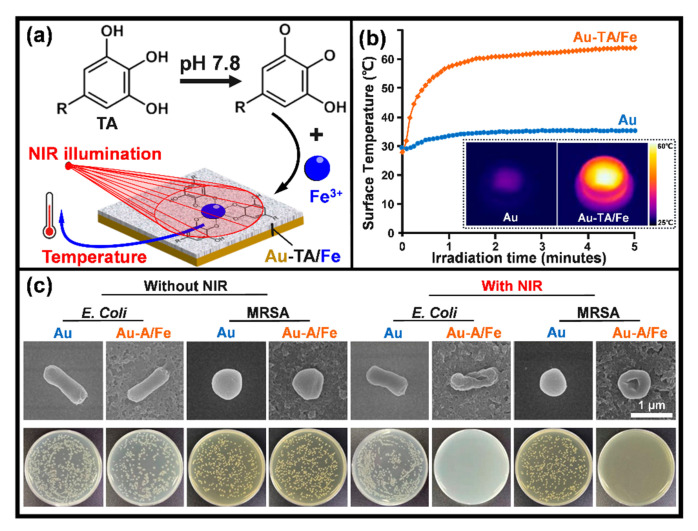
A photothermal antibacterial surface: (**a**) schematic illustration of the coordinated assembly of tannic acid (TA) and Fe^3+^ ions (iron chloride hexahydrate) on gold (can be other materials), yielding the Au-TA/Fe; (**b**) near-infrared (NIR) irradiation (808 nm, 2.2 W·cm^−2^) induced temperature changes on the material surface immersed in phosphate-buffered saline (PBS), with corresponding thermal images inserted; (**c**) SEM images of adherent bacteria (*E. coli* or MRSA) on materials surface with/without NIR irradiation (5 min), together with the typical photographs of bacterial colonies re-cultured from materials surface of different processing histories. Adapted from reference [178] with permission from the American Chemical Society.

Sterilizing materials’ surfaces with ultraviolet (UV) light is a well-established standard method that has been around for decades. Materials converting light energy to heat for local disruption of bacterial colonization, i.e., photothermal therapy, are promising alternatives to antibiotics that possibly circumvent the problem of resistance [176]. Typical reports in this direction are listed in Table 6. Gold nanoparticles have been studied widely because of their high efficiency of photothermal conversion via surface plasmon resonance in the near-infrared (NIR) region (in the range of 700–1100 nm) [177]. It was reported that a gold nanoparticle and phase-transitioned lysozyme hybrid film was able to kill 99% of adherent bacteria within 5 min under the illumination of a NIR laser [177]. Composite thin films were produced by coordinative assembly of tannic acid (TA) and iron ions (Fe^3+^) and yielded Au-TA/Fe (Figure 5a; the support can be other materials, rather than gold) [178]. These films exhibited high absorption and efficient light-to-heat convention under NIR irradiation (Figure 5b), as a result, they had efficient photothermal killing effects that disrupted 99% of adherent microbes, including both Gram-negative *E. coli* and Gram-positive MRSA strains (Figure 5c).

**Figure 6:**
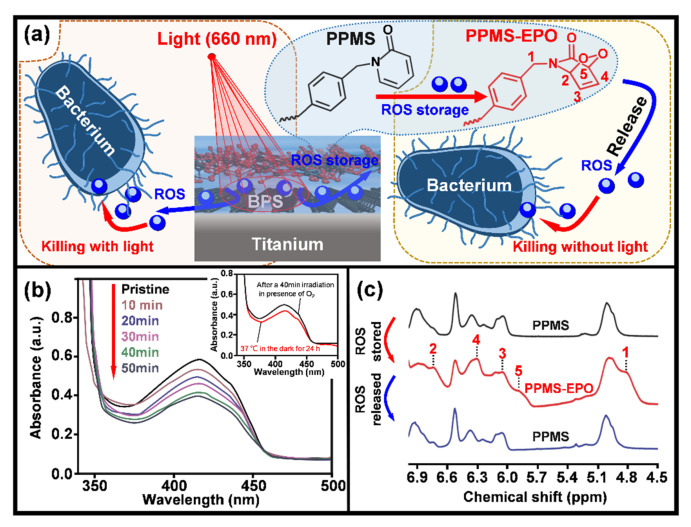
A photodynamic antibacterial material surface: (**a**) schematic illustration of the killing actions of the composite coating composed of black phosphorus nanosheets (BPS) and poly (4-pyridonemethylstyrene) (PPMS). Under light irradiation (660 nm, 0.5 W·cm^−2^), BPSs generate reactive oxygen species (ROS), which can directly act on bacterial cells or are stored by the coating itself through the transfer of PPMS into poly (4-pyridonemethylstyrene) endoperoxide (PPMS-EPO), yielding antibacterial activity in the dark (killing without light). (**b**) UV-vis spectra show the capability of ROS production in PPMS/BPS with the increasing irradiation duration in the air (20 °C, 660 nm, 0.5 W·cm^−^^2^). The insert shows the capability of ROS production by a PMMS-EPO/BPS (fabricated by illuminating the PPMS/BPS group for 40 min in presence of oxygen gas (O_2_) after being contained in the dark at 37 °C for 24 h. (**c**) ^1^H NMR spectra show the reversible structure change of PPMS and PPMS-EPO. Peaks corresponding to the endoperoxide ring and proton of endoperoxide were detected. Adapted from reference [181] with permission from John Wiley and Sons.

Materials absorbing light energy to produce reactive oxygen species (ROS) for oxidization of bacterial cell walls, yielding photodynamic therapies [179], are also developed for potential biomedical applications. Quantum dots are sensitive to blue light illumination and capable of producing singlet oxygen, which is a powerful ROS able to disintegrate bacterial cell walls [180]. It was reported that hydrophobic carbon quantum dots incorporated into various polymer matrices in form of thin films had significant activity against *S. aureus* under blue light irradiation [180]. An antibacterial coating composed of black phosphorus nanosheets (BPS) and poly (4-pyridonemethylstyrene) (PPMS) was coated on a titanium surface (PPMS/BPS) (Figure 6a) [181]. Under the stimulation of visible light (660 nm, 0.5 W·cm^−2^), the contained photosensitizer BPS released ROS (singlet oxygen), which was evidenced by the gradual decrease of UV absorption at 415 nm as the irradiation duration was prolonged to 50 min (Figure 6b). This was monitored by using 1, 3-diphenylisobenzofuran, which reacts with singlet oxygen to decrease the absorption around 415 nm [181]. In addition, the coating was good at the storage of the illumination-generated ROS via transforming PPMS to poly (4-pyridonemethylstyrene) endoperoxide (PPMS-EPO) (Figure 6a). The stored ROS can be released in the dark, mediating the “*killing without light*” capability of the coating. By illuminating in presence of oxygen gas (O_2_) for 40 min, the PPMS/BPS group was transferred into PMMS-EPO/BPS, which was able to release ROS even after being contained in the dark at 37 °C for 24 h (the insert in Figure 6b). A reverse transformation between pyridone and endoperoxide was evidenced by the arising 1H nuclear magnetic resonance (1H NMR) peaks corresponding to the endoperoxide ring and the proton of endoperoxide in PMMS-EPO (Figure 6c). Tan et al. demonstrated that the PPMS-EPO/BPS coating had an antibacterial rate (against E. coli and *S. aureus*) of over 99.0% under light illumination and around 70.0% without light irradiation [181]. Such designs can compensate those photo-based therapies for applications in implantable medical devices that lack light reach.

Materials having both photothermal and photodynamic activities are also developed for the disinfection of biomedical materials. Recently, a hydrothermally prepared nanorod array of titanium dioxide was demonstrated as an efficient photosensitizer for antibacterial applications [179]. In response to a NIR light (808 nm), the nanorods had both efficient light-to-heat conversion and ROS production properties, showing their excellent in vitro and in vivo antibacterial efficacy [179]. To endow titanium surface with both photothermal and photodynamic activities, the near-infrared fluorescent dye IR780 modified red phosphorus (has high photothermal conversion efficiency) films were developed [182]. In addition to the excellent compatibility with mammalian cells and normal tissue, the composite coatings demonstrated the synergistic effect of thermal and singlet oxygen in the eradication of *S. aureus* biofilms in vitro and in vivo [182]. Moreover, the thermal conversion activity of photosensitizers also can be applied to control the release of antibacterial agents for disinfection. Nano-structural molybdenum sulfide coating alone has high photothermal conversion efficacy that may induce hyperthermia capable of disintegrating bacterial envelopes [183]. This property of molybdenum sulfide can be used to control the release of antibacterial nitric oxide [184,185]. Typically, nano molybdenum sulfide assembled with heat-sensitive N,N′-di-sec-butyl-N,N′-dinitroso-1,4-phenylenediamine (as a nitric oxide donor) demonstrated a rapid antibacterial activity depending on nitric oxide release, yielding promising treatments for bacterial infections, even heat-resistant strain associated [184].

Moreover, antibacterial coatings responsive to other stimuli, such as temperature, electricity, and oxidative species, are also developed. Environmental temperature-responsive films based on poly(N-isopropylacrylamide) were developed to control the release of Ag NPs (at 37 °C) for antibacterial applications [186]. Triggered by an external electric field, a polypyrrole-doped polydimethylsiloxane coating was capable to release an antimicrobial drug loaded (crystal violet), making it a promising candidate for responsive antibacterial surfaces [187]. A branched poly(ethylene glycol)-poly(propylene sulfide) (PEG-PPS) polymer coating was found capable of actively releasing antibiotics (tigecycline or vancomycin) in response to an oxidative environment (ROS), which would occur adjacent to the infection site of a periprosthetic joint [188]. However, current reports on responsive antibacterial coatings normally are quite preliminary “*proof of concept*” studies; hence a lot of further efforts are needed to confirm their specific clinical scopes.

### 3.5. Cell-Selective Materials Surfaces

As aforementioned, an implant surface can be contaminated by pathogenic bacteria during surgery or during serving in the host. This requires the device to be highly selective over bacterial and mammalian cells, i.e., toxic to the adhesion of bacteria while compatible with the host cells. Here “*compatible*” includes two aspects, to be inert for a temporary device (for example a titanium bone plate) that does not stimulate rejections and to be bioactive for a permanent device (for example the implant-bone boundary for an artificial joint) that actively orchestrate tissue repair and integration in the host. We already know many surface features, such as surface composition (ion release), nanostructures, and wettability, that can determine the adhesion of cells to implantable medical device surface [189]. However, it is still hard to engineer a cell-selective surface on implantable medical devices because bacterial and mammalian cells share many mechanisms in adhesion, and the defense systems in the host are normally perturbed by placing the device. In the 1980s, Gristina first proposed the “*race for the surface*” concept [190], which suggested that the fate of an implantable device is a contest between bacterial adhesion and tissue integration to the device’s surface. If the race is won by tissue cells, then the device surface is normally in host protection from bacterial colonization and infections. Since then, various in vitro and in vivo methods were developed to simultaneously study biofilm formation and tissue integration on the same surface. Subbiahdoss et al. developed an in vitro method that allows the growth of both S. epidermidis (ATCC 35983) and osteosarcoma cells (U2OS) in a parallel plate flow chamber [191]. So the “*race for the surface*” can be evaluated by determining the number of adhering cells and the area per spread cell. By using this protocol, the race between *S. epidermidis* and U2OS cells on various polymers with different wettability was studied [192]. The results demonstrated that the interactions of U2OS cells with biomaterials were hampered by biofilm formation on the materials, and neither hydrophobic nor hydrophilic surfaces have the potential to help U2OS win the race. In contrast, the presence of integrin-active arginine-glycine-aspartic acid (RGD) peptide on biomaterials significantly compromised the negative effects of biofilm presence by increasing surface coverage of U2OS but detaching bacterial biofilms at elevated flow shear (5.6 s-1, phosphate-buffered saline) [193]. The competition for a poly(methylmethacrylate) surface between U2OS cells and highly virulent *S. aureus* or *P. aeruginosa* in presence of murine macrophages was also described by the research group [194]. The presence of *S. aureus* decreased the adherence of human osteogenic sarcoma (SaOS-2) or primary osteoblast (hOB) cells to the surfaces of titanium, polydimethylsiloxane, and polystyrene; on the other hand, the presence of either type of these human cells was also associated with a reduction of bacterial colonization to the material’s surface [195]. Martinez-Perez et al. described an in vitro approach for the study of the adherence of *S. aureus* and *S. epidermidis* in the presence of pre-osteoblastic cells (MC3T3-E1), which can be used for assessing the effects of surface coatings with antibacterial potentials [196]. By using a bilateral intramedullary rat model and injecting bacteria (*S. aureus*) into the tail vein after implant placement, the temporal interplay between host-cell adhesion and bacterial colonization was examined [197]. To determine the effects of hematogenous spreading of bacteria on infection subcutaneous implants (after healing of the implantation wound), rats were intravenously injected either with *S. aureus*, *S. epidermidis*, or *P. aeruginosa* 4 weeks after subcutaneous implantation of various biomaterials, including silicone rubber, polyethylene, polypropylene, poly(tetrafluoroethylene), poly(ethylene terephthalate), poly(methyl methacrylate), polyurethane, or glass [198]. The results demonstrated that late hematogenous infection of subcutaneous biomaterials does not occur in the rat, hence those reported late infections in humans were likely caused by perioperatively introduced pathogens [198], which is worthy of further studies. Reports covering biofilm formation on biomaterials surfaces lack often the differentiation between biofilm reducing and biofilm inhibitory effects [199]. Hence, the current biofilm methodologies used for judging the antibacterial effects of implant surfaces need to be critically revisited and if necessary revised and standardized. These reports show the significance of constructing cell-selective surfaces for implantable medical devices, as well as methods to evaluate the property.

Metallic ingredients are performing multiple functions in humans that can be building blocks leading to single-element-release mediated cell-selective surfaces. Zinc is known as a stimulus to the osteogenic function of bone cells and also an inhibitor of bacterial growth. Accordingly, zinc was loaded onto various titanium surfaces by using micro-arc oxidation [200], hydrothermal treatment [201], and ion implantation [202], demonstrating excellent antimicrobial and osteogenic properties. It is known that antimicrobial cobalt ions can induce hypoxia-like conditions [203]. By applying this feature of cobalt ions, antibacterial wound dressings with excellent capability for the promotion of angiogenesis and epithelialization were fabricated by Shi et al. [203]. Copper, in addition to its broad-spectrum bactericidal activity, was found to be capable of promoting osteoblast proliferation and bone formation [204]. It was reported that proper control of the content and release of copper in Ti-Cu alloys are capable of balancing the antibacterial and osteogenic properties of the metal implants [204].

The combinational release of antimicrobials and tissue-integration promoters is another pathway extensively studied to develop cell-selective implants. Low-molecular-weight polyethyleneimine is a cationic antimicrobial agent, and alendronate is a stimulus for new bone formation and osteointegration improvement [205]. They can be covalently conjugated onto ethanediamine-functionalized poly (glycidyl methacrylate) to construct titanium implants of both antibacterial and osteogenic properties [205]. Silver and hydroxyapatite are two typical materials with antibacterial and osteogenic properties, respectively. Recently, Fazel et al. developed a duplex process that sequentially employed micro-arc oxidation and hydrothermal treatment to decorate Ag NPs and hydroxyapatite nanocrystals on the surface of a porous Ti6Al4V substrate (fabricated by using selective laser melting), creating surfaces of both osteogenic and antibacterial properties [206]. Bacterial infection in burn wounds is common and fatal [207]. Many works have been done to develop wound dressings preventing bacterial infection and promoting wound healing. Porphyrin photosensitizer sinoporphyrin sodium has two macrocycles that show high efficiency against pathogenic bacteria via the production of ROS [208]. This dimeric photosensitizer was chosen to work with fibroblast growth factor in a carboxymethyl chitosan-sodium alginate matrix that has successfully suppressed the growth of bacteria and simultaneously accelerated the healing of bacteria-contaminated burn wounds in mice under mild photoirradiation (30 J·cm^−2^, 5 min) [208]. Due to inferior vascularization, poor re-epithelialization, and increased infection risk, treatment of diabetic wounds is considerably challenging and becomes a focus of cell-selective surfaces. The (11-mercaptoundecyl)-N,N,N-trimethylammonium (MTA) contains a quaternary ammonium cation that interacts strongly with the negatively charged cell membrane of microbes [209]. Together with the vascular endothelial growth factor, MTA can be conjugated into gold nanoparticles to produce dual-functional (antimicrobial and proangiogenic) dressings for treatments of diabetic wounds [209]. Ag NPs and pro-angiogenic deferoxamine were encapsulated in a pH-responsive hydrogel developed via double-crosslinking between chitosan quaternary ammonium salt and oxidized dextran-dopamine, achieving pH-sensitive feature in drug release and accelerated healing of infected diabetic wounds [160]. The antibacterial efficacy of hydroxypropyltrimethyl ammonium chloride chitosan is related to the substitution degree of its quaternary ammonium group [210]. This chitosan derivative cooperates with magnesium ions (magnesium chloride) in calcium alginate, yielding an antibacterial and angiogenic dressing for the treatment of infected diabetic wounds [210].

**Figure 7:**
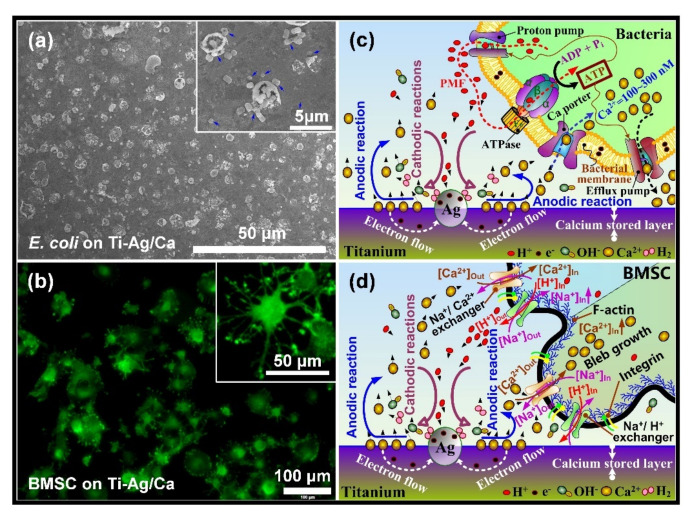
A cell-selective titanium surface: (**a**) SEM surface morphology of the microbes (*E. coli*) cultured for 24 h on titanium doped with both calcium and silver (Ti-Ag/Ca), with a high magnification image, inserted; (**b**) typical morphology of rat bone marrow stem cells (BMSCs) cultured for 1 h on Ti-Ag/Ca, with a high magnification image inserted; (**c**,**d**) potential mechanism underlying the actions of Ti-Ag/Ca on microbes and mammalian cells, respectively [215]. Reused with permission from the Royal Society of Chemistry.

Normally, the surface of a medical device will be in contact with body fluids, which are electrolytes that facilitate electrochemical reactions on the implant surfaces. These reactions can intervene in the adjacent microscale biological environments and subsequently determine the fate of the implants, giving alternative pathways to construct cell-selective surfaces. To be specific, the dissimilar phases in a metal likely have different electrode potentials, as a result, electrochemical corrosion (also known as internal galvanic corrosion) will occur when the material comes into contact with an electrolyte [211,212], just as the case that a metallic implant is in contact with the physiological fluid. Based on this mechanism, Cao et al. firstly proposed to control the antibacterial activity and improve the biocompatibility of Ag NPs by taking advantage of the chemical reactions stemming between nanosilver precipitates and the titanium matrix (Figure 2e) [143]. The antimicrobial activity of these immobilized (or embedded) Ag NPs was well retained in addition to their excellent compatibility with the functions of bone cells and bone formation [138,213,214]. The biological basis for such a cell-selective property is likely the difference in size and structure between the prokaryotic (bacteria) and eukaryotic cells (bone cells), which makes bacterial and mammalian cells respond differently to the proton-depleting reactions over Ag NPs decorated materials surface [143], i.e., proton depletion mediated by immobilized Ag NPs likely disrupts the transmembrane proton electrochemical gradient and inactivate the adenosine triphosphate synthesis, ion transport, and metabolite sequestration, ultimately lead the bacterial death (Figure 2e), while it catalyzes the activation of an integrin-mediated cascade of osteoblast differentiation in rat bone marrow stem cells and improving osteointegration of metal implants [144]. Such cell-selective effects can be further boosted via the co-doping of silver and calcium into titanium surfaces [215]. Silver and calcium were in situ introduced into the titanium surface by two synchronously operating cathodic arcs as aforementioned in Figure 2a. The dopped silver was condensed into nanoparticles (Ag NPs) on the substrate surface, while the calcium was intermixed with the titanium matrix, yielding a modified surface layer of approximately 30 nm in thickness (designated as Ti-Ag/Ca) [216]. *E. coli* (ATCC 25922) at a concentration of 10^6^ cfu·ml^−1^ and rat bone marrow stem cells (BMSCs) at a density of 10^4^ cells per ml were seeded onto the material surface and incubated at 37 °C for 24 h and 1 h, respectively. Serious bacterial cell disruption and distortion (Figure 7a) while accelerated spreading and coverage of BMSCs were detected (Figure 7b), demonstrating that Ti-Ag/Ca favored the functions of BMSCs and simultaneously acted against pathogenic bacteria, i.e., cell selectivity [215]. The cathodic and anodic reactions on Ti-Ag/Ca possibly disrupted the transmembrane proton-motive force (PMF) and the administration of calcium further stressed or even disordered the metabolic processes critical to bacterial maintenance (Figure 7c), showing the antibacterial activity as shown by Figure 7a. Additionally, the electrochemical reactions on Ti-Ag/Ca also possibly accelerated the proton extrusion of sodium-proton exchanger 1 (NHE1) and the calcium influx of sodium-calcium exchanger 1 (NCX1) in BMSCs (Figure 7d), showing enhanced membrane bleb nucleation, growth, and retraction as shown in Figure 7b. Moreover, the growth and retraction of membrane blebs can modulate cellular mechanics and promote the osteogenic differentiation of BMSCs, which is good to improve the osseointegration of titanium [215]. Such antibacterial surfaces are promising for orthopedic devices and dental implants, which are intended to be integrated into bone tissues.

In addition, topographical nanostructures, such as nanopillars [216,217,218], nanosheets [219], nanorod [220,221], and nano-roughness [222,223], with the efficacy of physical sterilization represent an innovative pathway toward antibacterial surfaces. Among these designs, nanopillars received the most attention because their puncture-based biocidal actions are material composition and bacterial species independent. The bactericidal mechanisms of such structures were already highlighted in a very recent review [224]. Additionally, there are several studies that demonstrated that titanium nanopillars with proper diameter, spacing, and height yield cell-selective surfaces. Hasan et al. fabricated nanopillars on a commercially pure titanium substrate (the surface appears black) using a reactive ion etching process [225]. They found that titanium nanopillars of about 1 μm in height have maximal bactericidal efficiency without compromising attachment and proliferation of human mesenchymal stem cells. Similarly, Ganjian et al. reported that titanium nanopillars with diameters of about 26 nm and lengths of about 1.1 μm have biocidal activity against Staphylococcus aureus and Escherichia coli, but murine preosteoblasts (*MC3T3-E1*) can attach and spread well [226]. Additionally, Modaresifar et al. recently concluded that the height and spatial organization are key factors contributing to the cell selectivity of the titanium nanopillars between *MC3T3-E1* and staphylococcus aureus [227]. These studies show that black titanium with nanopillars is a very promising material surface for orthopedic implants. However, further efforts are looking forward to confirming the cell selectivity of such a design in vivo.

### 3.6. Immune-Instructive Materials Surfaces

It is acknowledged that the high susceptibility of biomaterials to infections is not only related to bacterial contamination but also owed to the undesirable host responses which compromise the intrinsic immune capability in bacterial clearance [228,229]. The research of Zimmerli et al. revealed that 100 colony-forming units (CFU) of *S. aureus* were sufficient to cause infection to 95% of subcutaneous implants in pigs, whereas 10^5^-fold higher CFU did not produce any infection in the same subcutaneous model without alien implants [230]. Southwood et al. found that the surgical site of an arthroplasty implant became infected by contamination with 50 CFU of *S. aureus*. This concentration was 200 times lower than that causing infection in a surgical site without any foreign device [231]. Given this, the inherent immunomodulatory effects of implantable devices and their interactions with the host’s immunity system should be highlighted in developing advanced anti-infective biomaterials [232]. The immune system is generally divided into innate and adaptive arms, which cooperatively protect the host from bacterial infections [233]. The nonspecific nature of the former indicates its modulation potential yielding a broad spectrum against pathogenic bacteria [234]. The neutrophils and macrophages are key players mediating the innate immune response of the host at the cellular level. They have multiple actions against pathogenic bacteria, such as the production of reactive oxygen species (ROS) and reactive nitrogen species (nitric oxide), the release of granule proteins and neutrophil extracellular traps (NETs), cytokine expression of interleukins, and phagocytosis [235,236,237,238]; therefore, they are especially concerning to the biomaterial community.

The behaviors of neutrophil adhesion to biomaterials are determined by surface chemistry [239], topography [240], wettability [241], and stiffness [242]. Polystyrene and woven Dacron or Silastic induced neutrophil release of granule antibacterial products (are cationic peptides known as defensins) to create an environment hostile to phagocytic killing by neutrophils [243]. Polytetrafluoroethylene and Dacron promoted the production of ROS and mediated premature neutrophil death while polystyrene did not [239]. Furthermore, after being manually scratched with forceps, the polystyrene also induced the production of more reactive oxygen intermediates and rapid neutrophil death [240]. A rapid decrease in expression of L-selectin was detected within 16 min of neutrophil adhesion to titanium, and Fc gamma III receptor (CD16) expression dominated the initial adhesion (within 30 min) [244]. Human neutrophils rapidly adhered to sandblasted large-grit acid-etched titanium surfaces and released NETs [245], which are efficient actions of neutrophils limiting pathogenic spreading in a microbe-size-dependent manner [246]. Rough-hydrophilic titanium surfaces (produced by sandblasting and acid etching, and stored in a nitrogen environment) decreased the production of pro-inflammatory cytokines and enzymes as well as the formation of extracellular traps in adherent neutrophils [241]. In addition to the release of reactive oxygen radicals, neutrophils possibly enhance nitric oxide production in response to acute and chronic inflammation. The reaction of nitric oxide and reactive oxygen species readily generates peroxynitrite, which is a potent cytotoxic mediator [247]. It was demonstrated adherent neutrophils on polyethylene oxide-modified polyurethane produced lower amounts of nitric oxide; however, peroxynitrite formation did occur upon bacterial stimulation (*S. epidermidis*). This indicated that biomaterials can compromise neutrophil generation of nitric oxide, possibly diminishing the bacterial clearance capacity of the immune system and increasing the risk of DAIs [248]. Surface structures and stiffness have modulatory effects on macrophages. Surface-topography-induced changes in macrophages were examined in terms of polymer parallel gratings [248]. Such gratings, particularly of larger size, affected the adhesion, morphology, and cytokine secretion of macrophages. Adherent macrophages on both micro- and nanostructured silicon dioxide films did not increase the production of interleukin IL-6 or alter membrane mobility but had significantly greater phagocytic capacity than those on flat surfaces [249]. Macrophages are master regulators orchestrating host immune responses to biomaterials. Micropatterned surfaces (microgrooves/ridges and micropillars) did induce distinct gene expression profiles in human macrophages [250]. To be specific, micropillars (5–10 µm in diameter) were dominant in driving macrophage attachment to a polystyrene chip, and pillar size and spacing were critical in priming anti-inflammatory phenotype [251]. Hotchkiss et al. studied the surface roughness and wettability effects of titanium on macrophage activation and cytokine production. They found that smooth titanium induced inflammatory macrophage (M1) activation to express high levels of interleukins IL-1β, IL-6, and tumor necrosis factor alpha (TNF-α), while hydrophilic and rough titanium induced anti-inflammatory macrophage (M2) activation to release high levels of interleukins IL-4 and IL-10 [252]. Poly(ethylene glycol) based hydrogels with lower stiffness induced mild macrophage activation and a more typical mild foreign body reaction [253]. Similar results were found on polyacrylamide gels, i.e., stiff gels (323 kPa) prime pro-inflammatory macrophages with impaired phagocytosis while soft (11 kPa) and medium-stiff (88 kPa) gels prime anti-inflammatory and highly phagocytic phenotype [254]. These results were consistent with that reported by Previtera et al., who demonstrated that the production of pro-inflammatory mediators by macrophages was mechanically regulatable, namely stiff substrates enhanced proinflammation [255].

The immunomodulatory effects of antimicrobials attract extensive attention in addition to their biocidal activities. Silver-based coatings are commonly proposed for antibacterial applications; however, Croes et al. demonstrated that the electroplated silver on porous titanium was cytotoxic to neutrophils via releasing an excessive amount of silver ions and diminishing neutrophil phagocytic activity [256]. Diffusive Ag NPs were able to rapidly penetrate inside neutrophils and induce atypical cell death [257], which possibly inhibited neutrophil reactive oxygen production and subsequently impair the antibacterial efficacy of the innate immune system [258]. Other studies concluded that the immunomodulatory efficacy of diffusive Ag NPs was correlated with their ability to release silver ions [259]. Moreover, the immunomodulatory effects of engineered nanomaterials are chemistry-dependent. It was demonstrated that macrophages responded to diffusive nanoparticles by significantly increasing the generation of IL-6, nuclear translocation of nuclear factor-kappa B, induction of cyclooxygenase-2, and expression of TNF-α, with maximum prominent such pro-inflammatory responses detected in cells treated by diffusive Ag NPs, followed by aluminum, carbon black, and carbon-coated Ag NPs [260]. Moreover, zinc and copper are branded immunomodulatory and antimicrobial activities. Zinc plays multiple roles in the innate immune system, and zinc deficiency normally reduces the chemotaxis of neutrophils and the phagocytosis of macrophages [261]. Zinc oxide nanoparticles were coated on titanium by magnetron sputtering. This surface improved the antibacterial efficacy of macrophages and neutrophils in terms of phagocytosis and inflammatory cytokine secretion [262]. Copper-doped titanium oxide coatings (fabricated by micro-arc oxidation technique) induced high levels of inducible nitric oxide synthase activity and IL-6 release but low levels of IL-4 and IL-10 in macrophages to prime M1 phenotype, which exhibited enhanced phagocytosis and antibacterial efficacy [263]. Copper nanoparticles coated with polyetheretherketone (with a porous microstructure produced by sulfonation) by magnetron sputtering technique also were capable of polarizing macrophages to a pro-inflammatory phenotype with improved phagocytic ability toward MRSA [264].

**Figure 8:**
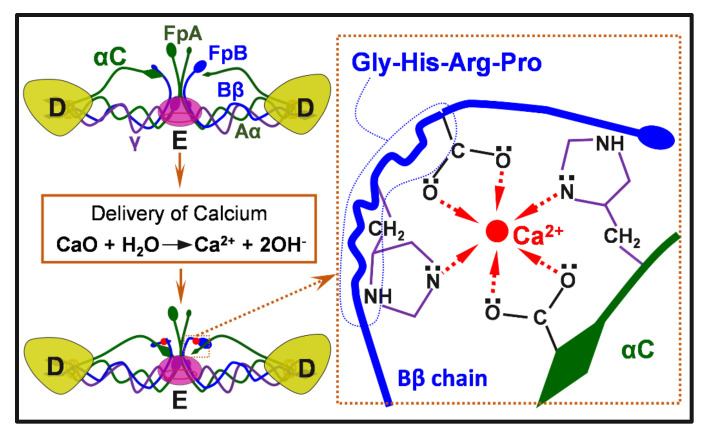
An antibacterial surface targeting the adsorption of fibrinogen: the calcium released by titanium turns the intramolecular interactions between αC regions and the amino-terminal of Bβ chains, and subsequently contributes to the exposure of the antibacterial peptide in fibrinogen. The Gly-His-Arg-Pro (Gly: glycine; His: histidine; Pro: proline; Arg: arginine) are the start sequences of the antibacterial peptide Bβ15–42 which locates at the N-terminal end of the β chain [16]. Reused with permission from the Royal Society of Chemistry.

This evidence gave us a comprehensive understanding of the biological actions of various silver-, zinc-, and copper-based materials, which are extensively concerned with developing antibacterial surfaces for medical devices [116,265]. However, they are normally passive studies that just demonstrate the immune-interfering actions of synthetic antibacterial materials, rather than active studies directly taking advantage of immunomodulatory biomaterials to construct antibacterial activity. In this respect, the immunomodulatory effects of essential metals, such as magnesium and calcium, should be appreciated. It was reported that high extracellular magnesium concentration can attenuate neutrophil activation by inhibiting the generation of superoxide radicals [266]. Calcium also plays a critical role in the regulation of pro-inflammatory functions of neutrophils, such as the release of superoxide anions, secretion of cytokine, formation of NETs, and phagocytosis [267]. Previously, we fabricated calcium-doped titanium (designated as Ti-Ca) by using a calcium plasma immersion ion implantation technique [268], which is similar to that shown in Figure 2a (just replace the silver cathode with pure calcium, and treat for 90 min with a 30 kV bias). Although in vitro tests demonstrated Ti-Ca was poor against bacterial colonization, the Ti-Ca implants survived the challenge of MRSA (ATCC 43300) in the tibia of rabbits and promoted osseointegration of titanium, while the pure titanium control (Ti) failed [256]. Very recently, we found that the locally delivered calcium by titanium can react with carboxy-terminal regions of the Aα chains and influence their interaction with the N-termini of Bβ chains in fibrinogen (a blood protein), which facilitates the exposure of the protein’s antimicrobial motifs, showing the surprising antimicrobial efficacy of calcium-doped titanium (Figure 8) [16]. This finding validates that the antibacterial surfaces can address the functions of the host rather than targeting directly the pathogenic bacteria, breaking the existing paradigm on minimizing DAIs. Fibrinogen adsorption is an essential process in the intrinsic immune responses of the human body to implantation operations, the aforementioned effect of calcium-doped titanium on fibrinogen adsorption brings further insight into the design of immunomodulatory biomaterials. In addition, the increased release ratio of magnesium/calcium from a magnesium alloy was found to prime the M2 phenotype of macrophages [269]. Since magnesium serves as a natural calcium antagonist [270], such synergistic effects of magnesium and calcium indicate a fruitful direction for the development of immunomodulatory antibacterial surfaces.

## 4. Directions to Improve the Quality of Antibacterial Reports

As demonstrated in Section 3, tremendous antibacterial designs have been proposed to treat DAIs. Despite a large number of studies carried out systematical in vitro and in vivo tests, clinical translation of these designs is limited. All the devices have their own “*intended use*”, which defines a primary function of an implantable medical device. For example, the primary functions of a wound dressing and dental implant are to help wound healing and promote osseointegration, respectively. Since not all the implantable medical devices are bound to a bacterial infection, antibacterial function, in our opinion, shall serve as a property secondary to those primary ones. This requires that the design, synthesis, and evaluation of implantable antibacterial surfaces should fit the needs of a specific “*intended use*”, which will help to clarify the exact biological environment that the material is intended to integrate [116,271]. This is crucial to choose proper routes for material synthesis, applicable parameters for material characterizations, and the right strategies for biological evaluations, enabling reproducible, comparable, and reusable results that will be consistent in clinical translation [271]. Unfortunately, many previous reports were at the stage of “*proof of concept*”; they did not conform strictly to a specific application. According to the papers we have screened, major flaws in our current reporting on developing antibacterial surfaces for implantable medical devices are (Table 7 includes some typical examples [18,272,273,274,275,276,277,278]) (1) many reports are lack of comprehensive understanding of the requirements of a specific application or even have no clear indication of use (Cases 2, 3, 7, and 8 in Table 7); as a result, it is hard to ensure the effectiveness and safety of such designs because the incidence and pathogens of DAIs are site-specific [66] and the biocompatibility of biomaterials is referred to specific applications [279]. (2) Some reports choose testing assays that do not closely relate to the intended use. For example, in Case 4, the authors reported a light-responsive material for antibacterial wound dressings; however, the effects of light illumination on mammalian cells are not considered either in vitro or in vivo. In Case 6, the intended application for the study is “*dental implant*”, which requires good osseointegration; nevertheless, only human gingival fibroblasts have been tested in vitro. (3) Some of the reports have flaws that possibly undermine the confidence in clinical applications. Typical features of these flaws are using unidentified bacterial sources (Cases 2 and 4 in Table 7), and changing experimental conditions during the study (for example, in Cases 5 and 6, the light irradiation parameters, power, duration, and onset changed between different in vitro and in vivo tests, which may mislead the follow-up studies). For the sake of safety, in addition to antibacterial tests, the tissue integration or compatibility of the designs shall be tested; however, this aspect was not considered in many current reports (in Case 8, for example, a light-responsive surface was proposed for disinfection; however, the effects of light illumination on tissue integration were not evaluated). Many studies are “*proof of concept*” reports (like Case 1), and some of them seem inconsistent according to their results. For example, the authors in Case 3 tried to report an antimicrobial surface with long-term efficacy, but the antibacterial effect was considered 5 days post-operation using a subcutaneous implant model in mice. Moreover, although a bacterial infection is possibly associated with multiple species (Case 8, Table 3), normally one bacterial strain is involved in tests; co-culture of mammalian cells with bacteria or co-culture of different bacterial strains are rare in current studies.

## 5. Summary and Outlook

Every DAI involves three participants, i.e., a device surface, pathogenic bacteria, and the host, which interact and interplay with each other and transform time dependently. That is why the onset of DAIs is uncertain and more DAIs are resistant to antibiotic treatments. Based on our growing knowledge of DAIs, the design paradigm toward implantable antibacterial surfaces is shifting to pursuing prolonged efficacy and being actively responsive, cell-selective, and immune instructive, experiencing a boom in advancing antibacterial surfaces (coatings) rapidly for various medical devices; nevertheless, clinical translation of these techniques is still rare. Since the incidence and associated bacterial strains of DAIs are site-specific, antibacterial designs for implantable medical devices shall conform with a specific intended use, which possibly promotes the clinical application of these technologies. Furthermore, the following aspects, in our opinion, are important to improve the quality of our reports in fundamental research. (1) Deepen interdisciplinary collaboration. The development of biomedical materials requires close collaboration in multiple disciplines, including materials sciences and engineering, biological sciences, medical sciences, etc. Researchers with a materials science and engineering background normally do not exactly know the experimental and reporting standards in biological and medical sciences; a closer collaboration will help to choose proper experimental assays and reduce the flaws in our publications, ensuring the impacts of our findings. (2) Publish in journals focusing on biomaterials science and engineering. Many academic journals are publishing biomaterials-associated studies; however, only those concentrated in biomaterials science and engineering are well equipped with experienced referees that can identify the flaws in the manuscript during the peer review process.

In addition, material designs targeting the adsorption processes of host proteins are a fruitful direction in developing implantable antibacterial surfaces. Spontaneous adsorption of proteins onto a biomedical device occurs seconds after its contact with body fluids, such as blood plasma, extracellular fluid, tears, saliva, and urine, depending on the specific intended use. Current efforts normally started both antibacterial tests and compatibility examinations at the cellular level regardless of the critical conditioning role of protein adsorption on the subsequent cell function and tissue integration. Blood plasma contains thousands of proteins that play key roles in diverse life activities, including signaling, transport, development, restoration, and disinfection [280]; however, plasma protein (fibrinogen) adsorbed to biomaterial surfaces is likely denatured into a pro-inflammatory state [281], which mediates foreign body reactions that contribute to DAIs [282]. Accordingly, control of protein adsorption in the host via material designs is possible to produce innovative antibacterial designs (reference No. 16 is one example) with a bright prospective for clinical applications, which is worthy of further efforts in the future.

## Figures and Tables

**Table 1:** Antibacterial surface registered for clinical studies *.

Active Ingredients	Devices	Phase	Locations	First Posted
Silver coating	Intravenous catheters	Not applicable	United States	25 August 2009
Antibiotics (minocycline and rifampin)	Antibacterial envelope for a cardiac implantable electronic device	Not applicable	United States	7 January 2010
Silver-based coating	Urinary catheter	Not applicable	United States	10 September 2012
Ionic silver	Wound dressings for a cardiac implantable electronic device	Phase 4	United States	24 May 2016
Silver-doped hydroxyapatite coating	Orthopedic implants (hip joint prostheses, intramedullary nails, and external fixator implants)	Not applicable	Turkey	17 November 2017
Gold-silver-palladium coating	Invasive devices (endotracheal tube, central venous catheter, and urinary catheter)	Phase 1, 2	Brazil	11 March 2019
Iodine	Barrier dressing for a cardiac implantable electronic device	Not applicable	Canada	19 October 2020
Antibiotic (gentamycin)	Platform wound device	Phase 4	United States	15 February 2021

* Data were obtained by searching for “*device infection*” in the “*Condition or disease*” field of the registered clinical studies conducted around the world on *ClinicalTrials.gov* (plus manual exclusion, as of 31 March 2022).

**Table 2:** Incidence of typical device-associated infections.

Device	Materials	Incidence	Reference
Ankle arthroplasty	Metals (titanium alloys), Ceramic, Polyethylene	2.4–8.9%	[25,26]
Hip arthroplasty	Metals (titanium alloys, stainless steel), Ceramics (alumina, zirconia), Polymers (polyethylene, polyetheretherketone), Composites	0.4–2.4%	[10,27,28]
Knee arthroplasty	Metals (titanium alloys, cobalt-chromium alloy), Ceramics (zirconia, titanium nitride), Polymers (polyethylene,)	1–2%	[10,29]
Breast implants	Silicone	1–10.2%	[30,31,32]
Vascular graft/endograft	Polytetrafluoroethylene, Polyethylene Terephthalate, Nitinol	0.16–6%	[33]
Cardiovascular electronic devices	Plastic polymers, Titanium, Teflon, Gold, Copper	0.9–7%	[34,35,36,37,38]
Cochlear implant	Teflon, Platinum-iridium alloy, Silicone, Titanium, Ceramics	1–8%	[39,40,41,42,43]
Brain stimulation implant	Stainless steel, Platinum, Titanium oxide, Iridium oxide	2–10%	[44,45,46]
Urinary catheters *	Natural rubber, Polyisoprene, Polymer ethylene vinyl acetate, Polytetrafluoroethylene, Hydrogel	0.1–13.7 cases per 1000 catheter-days	[47,48,49,50,51,52]
Cerebrospinal fluid shunts	Silicone rubber	1.9–27%	[53,54,55,56,57]
Internal fixation devices	Stainless steel, Cobalt-chromium alloys, Titanium alloys	7–32%	[58,59]
Dental implants	Titanium, Ceramics (zirconia, alumina)	6–47%	[60,61]

* The incidence of catheter-associated urinary tract infection is typically expressed as the number of infections per 1000 urinary catheter-days [52].

**Table 3:** Representative cases showing the latent period of DAIs.

Case	Devices	Latent Period (Post Insertion)	Pathogens	Causes	Reference
**1**	Alloplastic chin implant	45 years	/	After scratching herself (soft tissue degeneration due to aging)	[67]
**2**	Breast implant	Seven years	*Achromobacter xylosoxidans* (a pathogen that lives in wet soil)	Development of a chronic footsore (hematogenous spread from distant bacterial infection sites)	[68]
**3**	Breast implant	25 years	*Streptococcus viridans* (a pathogen that lives in the oral cavity)	After extensive dental treatment (hematogenous spread from distant bacterial infection sites)	[68]
**4**	Alloplastic implant	30 years	*Staphylococcus epidermidis*	Bacterial contamination years before identifying the infection (a symptom-free chronic infection; the pathogen escaped immune clearance and antibiotic treatments)	[69]
**5**	Orbital implant	30 years	*Cutibacterium acnes* (previously known as *Propionibacterium acnes*)	Bacterial contamination during the primary implantation (the pathogen can manifest for several decades)	[70]
**6**	Orbital implant	26 years (implant exposure 10 years before the presentation was documented)	*Propionibacterium acnes (renamed Cutibacterium acnes)*	Bacterial contamination during the primary implantation or implant exposure during scleral patch graft repair	[71]
**7**	Breast Implant	Five months	*Salmonella serogroup C*	Hematogenous seeding due to developing of diarrhea during a holiday travel	[31]
**8**	Generator for brain stimulation	Four months	Multispecies including the rare *Cupriavidus pauculus species* (an environmental Pathogen in “*water*”)	Penetration of contaminated water during participating in outdoor activities	[45]
**9**	Breast implant	Seven months	*Pasteurella canis* (a pathogen normally lives in the oropharyngeal commensal flora of cats and dogs)	Bacterial contamination from a patient-owned cat	[72]
**10**	Battery for brain stimulation	Two cases (Two years or 10 years)	*Staphylococcus aureus*	Chronic treatment of rheumatoid arthritis with methotrexate	[73]
**11**	Tibia Tenodesis Implant	Four and half months	*Nocardia nova* (a common environmental pathogen, rarely affects immunocompetent hosts)	Contamination of his tibial wound by the outside facility	[74]
**12**	Knee arthroplasty	4 months	*Listeria monocytogenes* (a facultative intracellular organism; commonly associated with deli meats and unpasteurized cheeses)	Consuming unpasteurized dairy products (an immunocompromised patient)	[75]
**13**	Hip arthroplasty	10 years	*Anaerobiospirillum succiniciproducens* (lives in the gastrointestinal tract of cats and dogs)	Breeding a dog (an immunocompromised patient)	[76]
**14**	Knee arthroplasty	Eight years	*Bartonella henselae* (a pathogen that induces acute infections but is hard to be diagnosed by culture)	A cat scratch	[77]
**15**	Cranioplasty implant	Two years and three months	No bacteria were cultured, but the infection was clinically evident	/	[78]
**16**	Shoulder prosthesis	Three years	*Staphylococcus* spp.	/	[79]

**Table 4:** Epidemiology of antibiotic-resistant DAIs.

Case	Resistant Pathogens	Implant	Latent Period	Reference
**1**	*Multidrug-resistant Acinetobacter baumannii*	Hip arthroplasty	12–25 days	[92]
**2**	*Methicillin-resistant Staphylococcus aureus (MRSA)*	Cardiac pacemaker	Nine years	[93]
**3**	*Clarithromycin-resistant Mycobacterium chelonae*	Breast implant	Four days	[94]
**4**	*MRSA*	Transvenous lead	Four years	[95]
**5**	*MRSA*	Ankle fracture fixation	Eight weeks	[96]
**6**	*MRSA*	Cranial implant	Three months	[97]
**7**	*MRSA*	Cochlear implant	Five months	[98]
**8**	*MRSA*	Pacemaker	Two months	[99]
**9**	*MRSA*	Breast Implant	Two days	[100]
**10**	*MRSA*	Laryngeal implant	More than one year	[101]
**11**	*Carbapenem-resistant Acinetobacter baumannii; Fluoroquinolone-resistant Enterobacter cloacae complex (AmpC overexpression)*	Internal fixation for an open proximal tibial fracture	Two months	[102]
**12**	*MRSA*	Pacemaker	Two years	[103]
**13**	*Multidrug-resistant Staphylococcus epidermidis*	Plates and wire cerclages for periprosthetic fractures	Three months	[104]
**14**	*Carbapenem-resistant Klebsiella pneumoniae*	Lumbar instruments,	Seven days	[105]
**15**	*MRSA*	The ventricular lead of an implanted defibrillator	Eight weeks	[106]
**16**	*Methicillin-resistant Staphylococcus haemolyticus*	Hip joint	Two years	[107]
**17**	*Ofloxacin-resistant staphylococcal endophthalmitis*	Intravitreal ozurdex implant	Three days	[108]
**18**	*MRSA*	Stent graft	Three days	[109]
**19**	*Methicillin-resistant Staphylococcus epidermidis*	Spinal instrumentation	7–88 days	[110]

**Table 5:** Representative reports on long-term antibacterial surfaces.

Active Ingredients	Intended Use (Substrates)	Effective Period	Reference
Tigecycline, Copper ions	Treatment for osteomyelitis (Alginate aerogel)	18 days	[121]
Vancomycin	Cement (Calcium phosphate)	168 days	[122]
(Z-)-4-bromo-5-(bromomethylene)-2(5H)-furanone	Dental implants (Titanium)	60 days	[123]
Silver/Zinc ions	An orthopedic and dental implant (Titanium)	180 days	[124]
Nanosilver	Bone implant (Polylactic acid fiber)	11 days	[125]
Honokiol	Remineralization of demineralized enamel (Poly(amido amine) (PAMAM) (Dendrimer)	24 days	[126]
Patchouli Essential Oil	Wound Dressing (Polyvinyl alcohol and chitosan)	2 days	[127]
Cetylpyridinium chloride	Endodontic sealers (Polyhydroxyethyl methacrylate trimethylolpropanetrimethacrylate)	48 days	[128]
Metallic silver	Hard tissue replacements (Titanium)	84 days	[129]
Copper	Orthopedics (Titanium)	14 days	[130]
Zinc/Copper	Cement (dicalcium silicate)	3 days	[131]
Amoxicillin	Wound dressing (Poly (e-caprolactone))	7 days	[132]
Chlorhexidine	Medical devices (not clear, 316L)	3 days	[133]
Silver ions	Orthopedic implants (Titanium)	189 days (silver release)	[134]
Nanosilver	Biomedicine (not clear)	7 days	[135]
Nanogold/Titania	Orthopedic implants (Titanium)	6 days	[136]
Nanosilver	Orthopedic implants (Titanium)	60 days	[137]
Silver nanoparticles	Orthopedic implants (Titanium)	60 days	[138]
Poly (poly (ethylene glycol) dimethacrylate)	Peritoneal dialysis catheters (Silicone)	30 days	[139]

**Table 6:** Representative reports on photo-responsive antibacterial surfaces.

Action: Active Ingredient	Light Parameter	Pathogens Tested	Intended Use	Reference
Heat: gold	NIR light	*E. coli*, *MRSA*	In vitro (not specific)	[177]
Heat: tannic acid and iron	NIR light	*E. coli*, *MRSA*	Not specific	[178]
Heat: titanium dioxide	NIR light	*E. coli*, *S. aureus*	Orthopedic/dental implants	[179]
Heat: carbon dots	Blue light	*S. aureus*	Not specific	[180]
ROS: black phosphorus	Visible light	*E. coli*, *S. aureus*	Implantable materials/device (not specific)	[181]
Heat and ROS: fluorescent modified red phosphorus	NIR light	*S. aureus*	Treatment for joint implants	[182]
Heat and Nitric oxide: molybdenum sulfide assembled with a nitric oxide donor	NIR light	*Ampicillin-resistant E. coli*, *heat-resistant E. faecalis*, and *S. aureus*	Wound repair (not specific)	[184]

**Table 7:** Typical flaws in our reports on antibacterial surfaces.

Case	Antibacterial Designs	Bacterial Strain (In Vitro)	Mammalian Cells Line (In Vitro)	In Vivo Tests	Intended Use	Reference
**1**	**Cell-selective:** Coating titanium nanowires with poly (ethyl acrylate) to organize fibronectin and deliver BMP-2	*P. aeruginosa* (ATCC 27853); cultured for 24 h	Primary human mesenchymal stem cell (MSCs); co-culture with bacteria	*None*	Orthopedic implant	[272]
**2**	**Cell-selective:** Ion release by Magnesium hydroxide	*S. aureus* (*unidentified source*); E. coli (*unidentified source*)	Mouse *MC3T3-E1* pre-osteoblasts	Rat femoral condyle defect model; *Placed in for 7 days to examine the dis-infective effects. Placed in for 4 weeks to evaluate the osteogenic property*	*Not specific*	[273]
**3**	**Long-term efficacy:** salt-responsive polyzwitterionic brushes on a nanopatterned surface	*P. aeruginosa* (BNCC 337005); *Escherichia coli* (ATCC 25922)	Rabbit red blood cells (2 h- incubation); *L929* fibroblasts (cultured for 24 h)	Subcutaneous implant model in mice; *Placed in for 5 days*	*Not specific*	[274]
**4**	**Light-responsive** (808 nm laser irradiation, 1 W/cm^2^, 5 min): Photosensitive gelatin methacryloyl incorporated with 4-octyl itaconate bearing black phosphorus	*S. aureus* (*unidentified source*); E. coli (*unidentified source*); *The onset of light irradiation is not clear*	Human umbilical vein endothelial cells; *The effect of light illumination on the cell function was not clear (No data presented)*	Rat type I diabetes model (14 days); *The onset for light irradiation is not clear*	Wound dressing	[275]
**5**	**Light-responsive** (1060 nm laser, 0.3 W/cm^2^, 0.6 W/cm^2^, and 0.9 W/cm^2^): Yb and Er-doped titanium dioxide nano-shovel/quercetin/L-arginine coatings	*S. aureus* (ATCC 29213);Light illumination (*0.6 W/cm^2^,15 min*)	Osteosarcoma cells (Saos-2, *light irradiation at 0.9 W/cm^2^ for 10 min*); Human umbilical vein endothelium cells (*light irradiation at 0.6 W/cm^2^ for 10 min*); Bone marrow mesenchymal stem cells (*light irradiation at 0.6 W/cm^2^ for 10 min*)	Tumor-bearing mouse model (l*ight irradiation at 0.9 W/cm^2^ for 10 min and performed every other day*); Mice tibia infection model (*light irradiation at 0.6 W/cm^2^ for 15 min and performed one day after surgery*); Mice tibia osteogenic model (*light irradiation at 0.6 W/cm^2^ for 15 min and performed one day after surgery; samples collected 4 weeks after surgery*)	Bone implants	[18]
**6**	**Light-responsive** (808 nm laser irradiation): TiO_2_/TiO_2−x_ metasurface	E. coli (ATCC 25922); *S. aureus* (ATCC 43300); *illuminated at 0.5 W/cm^2^ for 10 min*	Human gingival fibroblasts; *light illumination at 0.5 W/cm^2^ for 10 min*	Subcutaneous model in rats; *light illumination at 1.4 W/cm^2^ for 10 min after surgery*	Dental implant	[276]
**7**	**Immune-instructive:** polydopamine functioned and antimicrobial peptide plasmid (LL37 plasmid) loaded porous zeolitic imidazolate framework-8 (ZIF8) in 3D-Printed Scaffolds	MRSA (ATCC 43300); *E. coli* (ATCC 25922)	MC3T3 cell; *The material effects on immune systems are not considered*	Murine quadriceps muscle infection model (MRSA injected after scaffold placement)	*Not specific*	[277]
**8**	**Light-responsive** (808 nm laser irradiation, 2 W/cm^2^, 10 min): self-assembly of copper sulfide nanoparticle and reduced graphene oxide on anodized titanium	*S. aureus* (ATCC 29213); *E. coli* (ATCC 25922); *Light irradiation after inoculation*	Mice bone marrow stromal cells; *The effect of light illumination on the cell function is not clear (No data presented)*	Disinfection in rats (7 days); Osteogenic property in rats (8 weeks); *The effect of light illumination on osteogenesis was not clear (No data presented)*	*Not specific*	[278]
